# 1743. Health Care Resource Utilisation (HCRU) Due to Respiratory Syncytial Virus (RSV) Before the Age of Two in England

**DOI:** 10.1093/ofid/ofad500.1574

**Published:** 2023-11-27

**Authors:** Maria João Fonseca, Mathieu Bangert, Rolf Kramer, Lorena Cirneanu, Richard Hudson

**Affiliations:** IQVIA, Porto, Porto, Portugal; Sanofi Vaccines, Lyon, France, Lyon, Rhone-Alpes, France; Sanofi Vaccines, Lyon, France, Lyon, Rhone-Alpes, France; IQVIA, Porto, Porto, Portugal; Sanofi, London, England, United Kingdom

## Abstract

**Background:**

RSV is responsible for 33 million lower respiratory tract infections (LRTI) worldwide. The highest burden occurs in infants < 1 year of age during RSV season, which is from October to February in England. Due to lack of routine laboratory testing, its true disease burden is unknown. This study aimed to estimate the respiratory related primary and secondary care healthcare resource utilisation (HCRU) of RSV admissions before the age two in England.

**Figure:**
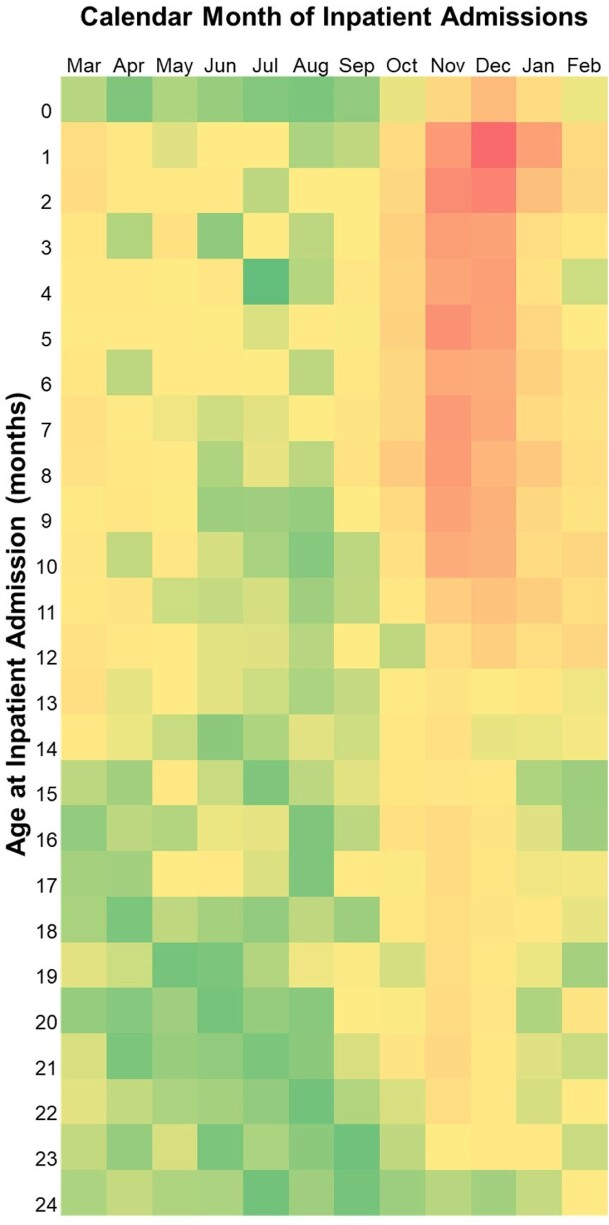

**Methods:**

This retrospective cohort study used Clinical Practice Research Datalink (CPRD)-Hospital Episode Statistics (HES) linked data to establish a birth cohort of all infants born between 01/03/2015 and 28/02/2017 (n=449,591). Case cohorts included infants under two with a hospital admission of: 1) confirmed RSV (n=4,813), 2) bronchiolitis (n=22,913), 3) any RTI (n=56,871). Each case cohort was compared with all birth cohort infants without the corresponding admission (comparative cohorts). Respiratory related HCRU (primary care, outpatient, accident and emergency [A&E], and inpatient admissions) were estimated in the month prior to and up to 24 months following the first admission in each cohort.

**Results:**

The RSV-coded cohort had the highest HCRU overall, mean (SD) of 5.1 (4.8) admissions vs. 1.5 (2.4) in its comparative cohort (71% difference). This difference was largely due to A&E and inpatient admissions. Comparable results were found for bronchiolitis-coded and RTI-coded cohorts. The highest rates of inpatient admissions were observed for the RSV-coded case cohort, and these occurred during the calendar months of November and December. Inpatient admissions were largely among infants up to 12 months of age and included infants that were born both before and during the season (Figure 1).

**Conclusion:**

Infants with an RSV admission had higher HCRU than infants without. Most hospitalisations occurred in November and December when services are at their most stretched. During the first year of life, the HCRU rates observed for infants born before and in the season were equally very high, emphasizing the need for preventative strategies to all infants entering their first RSV season. **Funding:** This research was funded by collaborative agreement with Sanofi / AstraZeneca and is part of the RODEO research program for RSV disease.

**Disclosures:**

**Maria João Fonseca, n/a**, Sanofi: Advisor/Consultant **Mathieu Bangert, PhD**, Sanofi: Staff member **Rolf Kramer, n/a**, Sanofi: Stocks/Bonds **Richard Hudson, n/a**, Sanofi: I am an employee of Sanofi|Sanofi: Stocks/Bonds

